# Prediction of Protein-Protein Interaction Sites by Random Forest Algorithm with mRMR and IFS

**DOI:** 10.1371/journal.pone.0043927

**Published:** 2012-08-28

**Authors:** Bi-Qing Li, Kai-Yan Feng, Lei Chen, Tao Huang, Yu-Dong Cai

**Affiliations:** 1 Institute of Systems Biology, Shanghai University, Shanghai, People’s Republic of China; 2 Key Laboratory of Systems Biology, Shanghai Institutes for Biological Sciences, Chinese Academy of Sciences, Shanghai, People’s Republic of China; 3 Shanghai Center for Bioinformation Technology, Shanghai, People’s Republic of China; 4 Beijing Genomics Institute, Shenzhen, People’s Republic of China; 5 College of Information Engineering, Shanghai Maritime University, Shanghai, People’s Republic of China; 6 Department of Genetics and Genomic Sciences, Mount Sinai School of Medicine, New York City, New York, United States of America; Semmelweis University, Hungary

## Abstract

Prediction of protein-protein interaction (PPI) sites is one of the most challenging problems in computational biology. Although great progress has been made by employing various machine learning approaches with numerous characteristic features, the problem is still far from being solved. In this study, we developed a novel predictor based on Random Forest (RF) algorithm with the Minimum Redundancy Maximal Relevance (mRMR) method followed by incremental feature selection (IFS). We incorporated features of physicochemical/biochemical properties, sequence conservation, residual disorder, secondary structure and solvent accessibility. We also included five 3D structural features to predict protein-protein interaction sites and achieved an overall accuracy of 0.672997 and MCC of 0.347977. Feature analysis showed that 3D structural features such as Depth Index (DPX) and surface curvature (SC) contributed most to the prediction of protein-protein interaction sites. It was also shown via site-specific feature analysis that the features of individual residues from PPI sites contribute most to the determination of protein-protein interaction sites. It is anticipated that our prediction method will become a useful tool for identifying PPI sites, and that the feature analysis described in this paper will provide useful insights into the mechanisms of interaction.

## Introduction

Proteins play critical roles in nearly all biological events by interacting with other proteins, compounds, RNA and DNA. Understanding the characteristics of interaction sites is basic to understanding the molecular recognition process. Proteins rarely act in isolation and often exert their functions by being part of a large molecular network, with roles coordinated via complicated regulatory networks of protein-protein interactions (PPI). Thus, protein complexes rather than individual components would determine the behavior of a biological system. PPI are crucial to nearly all aspects of cellular functions, including regulation of signaling and metabolic pathways, protein synthesis, DNA replication and gene translation, as well as immunological recognition [1]. In particular, identifying the binding sites between two interacting proteins would provide valuable clues for understanding and determining the functions and structures of protein complexes, for facilitating the identification of pharmacological targets and ultimately for drug design. Therefore, predicting the interaction sites is of great significance.

Hitherto several methods have been proposed to predict PPI sites and they can be roughly divided into three classes based on the features used. Methods in the first class are based only on sequence information [2], [3], [4]. The same dataset including 1,134 chains in 333 complexes with 59,559 contacting residues were used in Ofran and Rost’s studies [3], [4]. In their 2003 study [3], when 70% of their predictions were accurate, they correctly predicted at least one interaction site in 20% of the complexes (66/333). In 2007, they proposed another method and improve the prediction accuracy [4]. Methods in the second class integrate secondary structural information and sequence information [5], [6]. The work of Wang et al. [5] was based on a non-redundant data set of heterodimers consisting of 69 protein chains and achieved a sensitivity of 66.3%, a specificity of 49.7%, an accuracy of 0.654 and a correlation coefficient of 0.297. Zhou and his coworkers [6] constructed a predictor trained on 615 pairs of nonhomologous complex-forming proteins and tested on a different set of 129 pairs of nonhomologous complex-forming proteins. With this method, 70% of the 11,004 interface residues were correctly predicted. Methods in the third class use 3D structural information or integrated 3D structure with sequence information for the prediction [7], [8], [9]. Aytuna et al. [7] proposed an algorithm, which was run on a template dataset of 67 interfaces and a sequentially non-redundant dataset of 6,170 protein structures. The majority of the predicted 62,616 potential interactions were verified in public databases including Database of Interacting Proteins, Biomolecular Interaction Network Database and PDB. In the work of Sikic et al., they used the same dataset as those in Ofran and Rost’s studies [3], [4]. In this study, the sequence-based prediction achieved a precision of 84% with a 26% recall. After combination with structural information, the prediction performance increases to a precision of 76% and a recall of 38% [9]. Based on different kinds of features, several machine learning approaches have been proposed to predict protein-protein interaction sites, such as neural networks [3], [10], support vector machines (SVM) [11], [12], [13], Bayesian network [14], hidden Markov models (HMM) [15] and conditional random fields [16]. In Li et al. [13], the best SVM model trained was tested on a set of 50 randomly selected proteins. The sensitivity, specificity, and MCC for the prediction of the core interface residues were 60.6%, 53.4%, and 0.243, respectively. In Bradford et al. [14], a Bayesian network was used to predict protein-protein binding sites with a success rate of 82% on a benchmark dataset of 180 proteins. In a study of Li and his coworkers [16], when 1276 non-redundant hetero-complex protein chains were used as training and test set, the best precision, recall, accuracy and MCC were 0.536, 0.595 0.692 and 0.328, respectively.

Although great progress has been made, the problem of predicting interaction sites is still far from being solved. Several challenges remain to be overcome. Firstly, specific biological characteristics for precisely identifying protein-protein interaction sites are not fully elucidated [17]. It seems impossible for any single parameter to distinguish interaction interfaces from other surface patches [11]. Therefore, many studies tried to combine multiple characteristics to predict protein-protein interaction sites. Secondly, existing methods often utilize information derived directly from amino acid sequences to predict protein-protein interaction residues, which is not sufficient to excavate all important information [18]. Thirdly, a skewed class distribution problem exists ubiquitously in the prediction of protein interaction sites. The quantity of interacting sites of a protein is usually much less than that of non-interacting sites [17]. Such an imbalance tends to cause overfitting and poor performance, especially affecting data in the interacting class.

In this study, we developed a novel method to predict protein-protein interaction sites based on a Random Forest (RF) algorithm with a Minimum Redundancy Maximal Relevance (mRMR) method followed by incremental feature selection (IFS). We not only incorporated features of physicochemical/biochemical properties, sequence conservation, residual disorder, secondary structure and solvent accessibility, but also included five 3D structural features to predict protein-protein interaction sites. Feature analysis shows that structural features such as Depth Index (DPX) and surface curvature (SC) contribute most to the prediction. It is also shown via the site-specific feature analysis that the features of individual residues from PPI sites contribute most to the determination of protein-protein interaction sites.

## Materials and Methods

### Dataset

Our protein-protein interaction (PPI) datasets were retrieved from the database of three-dimensional interaction domains (3did) (http://3did.irbbarcelona.org) [19], collecting protein interactions for which high-resolution three-dimensional structures are known. 3did exploits the availability of structural data to provide molecular details for interactions between two globular domains. We deleted the sequences whose lengths were less than 50 and then deleted the homologous sequences in the original dataset of the 120,622 chains with a threshold of 25% identity measured by CD-HIT [20]. Finally, we obtained 6,488 chains specified by 3,353 PDB structures.

Then we extracted 21-residue protein segments centered on the annotated protein-protein interaction residue, with 10 residues upstream and 10 residues downstream of the interaction site. For the peptides with lengths less than 21 amino acid residues, we complement it with “X”. We regarded the peptides centered on the annotated interaction as positive data, while the other non-interaction peptides are termed negative data. We obtained in total 104,802 positive samples and 180,698 negative samples. After removal of the peptides centered on a buried residue, 38,446 positive samples and 85,340 negative samples remained. We then deleted the homologous peptides in the positive and negative samples with a threshold of 40% identity measured by CD-HIT [20], and obtained 13,427 positive samples and 12,429 negative samples.

### Features

#### PSSM conservation scores

Evolutionary conservation play an important role in biological analysis. A more conserved residue within a protein sequence may indicate that it is more important for the protein function and thus under stronger selective pressure. We used Position Specific Iterative BLAST (PSI BLAST) [21] to measure the conservation status for a specific residue. A 20-dimensional vector was used to denote probabilities of conservation against mutations to 20 different amino acids for a specific residue. For a given peptide, all such 20-dimensional vectors for all residues composed a matrix called the position specific scoring matrix (PSSM). In this study, we used the PSSM conservation score to quantify the conservation status of each amino acid in a protein sequence.

#### Amino acid factors

Since each of the 20 amino acids has different and specific properties, the composition of these properties of different residues within a protein can influence the specificity and diversity of the protein structure and function. AAIndex [22] is a database containing various physicochemical and biochemical properties of amino acids. Atchley et al. [23] performed multivariate statistical analyses on AAIndex and transformed AAIndex to five multidimensional and highly interpretable numeric patterns of attribute covariation reflecting polarity, secondary structure, molecular volume, codon diversity, and electrostatic charge. We used these five numerical pattern scores (denoted as “amino acid factors”) to represent the respective properties of each amino acid in a given protein.

#### Disorder score

Protein segments lacking fixed three-dimensional structures under physiological conditions play important roles in biological functions [24], [25]. The disordered regions of proteins allow for more modification sites and interaction partners, as well as usually containing PTM sites, sorting signals, and protein ligands. Therefore such regions are quite important for protein structure and function [24], [26], [27]. In this study, VSL2 [28], which can accurately predict both long and short disordered regions in proteins, was used to calculate a disorder score to denote the disorder status of each amino acid in a given protein sequence.

#### Secondary structure and solvent accessibility

The protein structures playing important roles in protein function and the post-translational modifications of specific residues may be influenced by the solvent accessibility of the relevant residues. In our study, we also used the structural features including secondary structure and solvent accessibility to encode the peptides. Of these features, the solvent accessibility and secondary structure, were predicted by the predictor SSpro4 [29]. SSpro4 designates the secondary structural property of each amino acid as ‘helix’, ‘strand’, or ‘other’, encoded as 100, 010 and 001 respectively, and terms the solvent accessibility of each amino acid ‘buried’ or ‘exposed’, encoded as 10 and 01 respectively. Since buried residues almost never occur in a protein interface, we removed all the peptides centered on a residue predicted to be buried in both positive and negative samples [6], [30].

#### Protrusion index and depth index

It has been shown that geometrical properties of the protein surface can influence protein-protein interactions [31]. In our study, we also used 3D structural features including Protrusion Index (CX) and Depth Index (DPX) to encode the peptides. These features were predicted by the Protein Structure and Interaction Analyzer (PSAIA) from PDB data. PSAIA was developed to compute geometric parameters for large sets of protein structures to predict and investigate protein-protein interaction sites [32].

### Accessible Surface Area, Molecular Surface Area and Surface Curvature

Research has shown that stability and solubility of proteins are determined by the manner in which elements of the macromolecular surface interact with solvent and small solutes in solution. Therefore, macromolecular surface is one of the most important factors in analyzing macromolecular structure and function. In this study, we also considered three other 3D structural features including accessible surface area (AS), molecular surface area (MS) and surface curvature (SC) to encode the peptides. These features were predicted by Program SurfRace from PDB data [33].

#### The feature space

For each residue of a protein segment, we incorporated 34 features, including 20 features of the PSSM conservation score, 1 disorder feature, 5 features of AAFactor, 3 features of secondary structure and 5 3D structural features from PDB data. Overall, for the 21-residue peptide there are a total of 34×21 = 714 features. For 21-residue peptides complemented with “X” residues, all features of these “X” residues are denoted as 0. To determine whether 3D structural features can improve the prediction performance, we also constructed another dataset without the 5 3D structural features. Therefore, there are a total of 29×21 = 609 features for this dataset.

#### mRMR method

We used the Minimum Redundancy Maximal Relevance (mRMR) method to rank the importance of the features [34]. The mRMR method ranks features based on both their relevance to the target and the redundancy between features. A smaller index of a feature denotes that it has a better trade-off between maximum relevance to the target and minimum redundancy.

Both relevance and redundancy were quantified by mutual information (MI), which estimates the extent to which one vector is related to another. The MI equation is defined as:
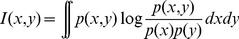
(1)In equation (1),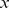, 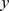
are vectors, 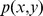
is their joint probabilistic density, and 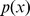
and 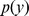
are the marginal probabilistic densities.


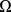
is used to denote the entire feature set. 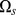
is used to denote the already-selected feature set containing m features and 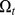
is used to denote the to-be-selected feature set containing n features. The relevance 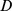 between the feature *f* in 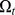 and the target 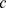 can be calculated by:
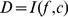
(2)The redundancy 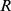 between the feature 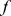
in 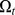 and all the features in 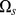 can be calculated by:
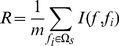
(3)To determine the feature 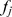 in 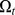
with maximum relevance and minimum redundancy, the mRMR function combines equation (2) and equation (3) and is defined as:
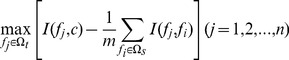
(4)The mRMR feature evaluation would continue N rounds when given a feature set with N (N = m+n) features. After the mRMR feature evaluation, a feature set 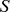 is obtained:
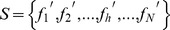
(5)In this feature set 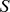, the index h of each feature indicates at which round the feature is selected. The smaller the index h, the earlier the feature satisfies equation (4) and the better the feature is.

#### Prediction engine and assessment

In the current study, Random Forest was adopted as the prediction engine and operated with the default parameters. Random Forest is an ensemble predictor that consists of a certain number of decision trees. To classify a new query sample coded by an input vector, the sample is placed into each of the trees in the forest. Each decision tree provides a predicted class. The class with the most votes will be put forward as the predicted class of the random forest. The detailed procedure can be found in Ref. [35]. Ten-fold cross-validation was used to evaluate the performance of our method [36]. TP denotes true positive. TN denotes true negative. FP denotes false positive and FN denotes false negative [37]. To evaluate the performance of our protein-protein interaction site predictor, the prediction sensitivity (also known as recall), precision, specificity, accuracy and MCC (Matthews correlation coefficient) were calculated as shown below:
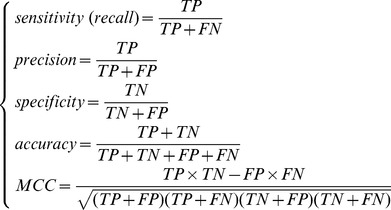
(6)As we know, the precision-recall curve is often used to evaluate the classifier’s performance [38]. A classifier makes its prediction for each sample based on a threshold, “k”, that is often defined as 0.5. For a classifier and a certain threshold, the predicted results obtained by this classifier can be represented by a confusion matrix, including four entries: TP, FN, FP, and TN. Thus, we can obtain precision and recall for different thresholds “k”, thereby plotting a point with precision as its Y-axis and recall as its X-axis in a coordinate system [39]. The obtained curve is termed the “precision-recall curve”.

The default parameters of the Random Forest are listed below:

-c <class index>
2. Sets index of class attribute (default: last).
-x <number of folds>
4. Sets number of folds for cross-validation (default: 10).
-s <random number seed>
6. Sets random number seed for cross-validation or percentage split (default: 1).
-S
8. Seed for random number generator. (default 1).
-depth <num>
10. The maximum depth of the trees, 0 for unlimited. (default 0).
-threshold-label <label>
12. The class label to determine the threshold data. (default is the first label).

#### Incremental Feature Selection (IFS)

Based on the ranked features obtained by the mRMR approach, we used the IFS [40], [41] approach to determine the optimal number of features. During the IFS procedure, features in the ranked feature set were added with a stepwise of 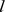 features from higher to lower rank. A new feature set was formed when 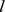 features had been added. Thus 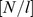 feature sets would be composed for N ranked features. The i-th feature set is:
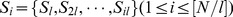
(7)where N denotes the total number of features in the original dataset and *l* (step) is a positive integer. In this study 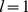. For each of the [N/l] feature sets, an RF classifier was constructed and examined using the 10-fold cross-validation on the benchmark dataset. By doing so we obtained an IFS table with one column for the index i and the other four columns for the prediction accuracy, sensitivity, specificity and MCC, respectively. Thus, we could obtain the optimal feature set (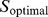), with which the predictor would yield the best prediction performance.

## Results and Discussion

### The mRMR Result

Listed in Information S1 are two outcomes obtained by running the mRMR software: one is a MaxRel feature table that ranks the 714 features according to their relevance to the class of samples; the other is called the mRMR feature table that lists the ranked 714 features according to mRMR criteria. In the mRMR feature table, a feature with a smaller index implies that it is more important for PPI site prediction. Such a list of ranked features has been used in the following IFS procedure for optimal feature set selection.

### IFS Results

**Figure 1:**
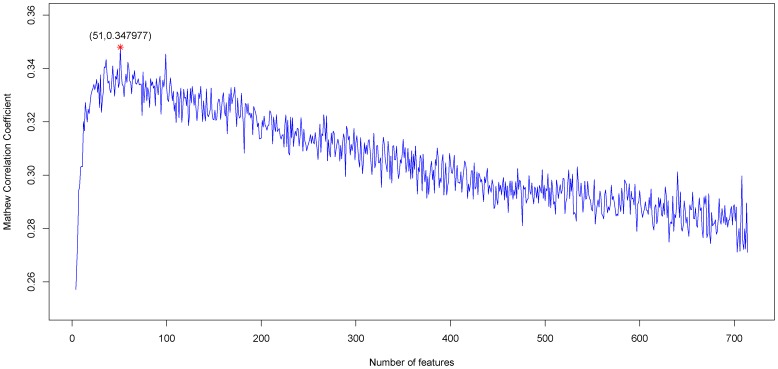
A plot to show the change of MCC values versus feature numbers. The IFS curves were drawn based on the data in Information S2. The MCC value reached a peak when the number of features was 51. The 51 features thus obtained were used to form the optimal feature set for the PPI site predictor.

Referring to IFS in Materials and Methods, by setting 714 for N and 1 for the feature-increasing gap, 714 individual predictors corresponding to 714 feature subsets were constructed to predict the PPI sites in the sequence samples. Listed in Information S2 are the rates of prediction accuracy, specificity, sensitivity and MCC obtained by each of the 714 predictors. Shown in Fig. 1 is the IFS curve plotted based on the data in Information S2. From Fig. 1 we can see that the predictor achieved a maximum of MCC equaling 0.347977 when 51 features were included. These 51 features were deemed as the optimal feature set of our classifier. With such a classifier, the prediction sensitivity(recall), precision, specificity and accuracy were 0.789975, 0.653060, 0.546625 and 0.672997 respectively (Table 1). The optimal 51 features are given in Information S3. Hereafter, all the analyses are based on these 51 optimal features.

**Table 1:** Comparison of prediction performances of different methods.

Method	Sn	Pr	Sp	Ac	MCC
Without 3D structural features	0.720340	0.591741	0.463110	0.596689	0.190073
With 3D structural features	0.789975	0.653060	0.546625	0.672997	0.347977
Sikic et al.’ method [9]	0.782751	0.634470	0.512833	0.653001	0.307795

Sn: sensitivity (recall).

Pr: precision.

Sp: specificity.

Ac: accuracy.

MCC: Matthews correlation coefficient.

### Feature Analysis

**Figure 2:**
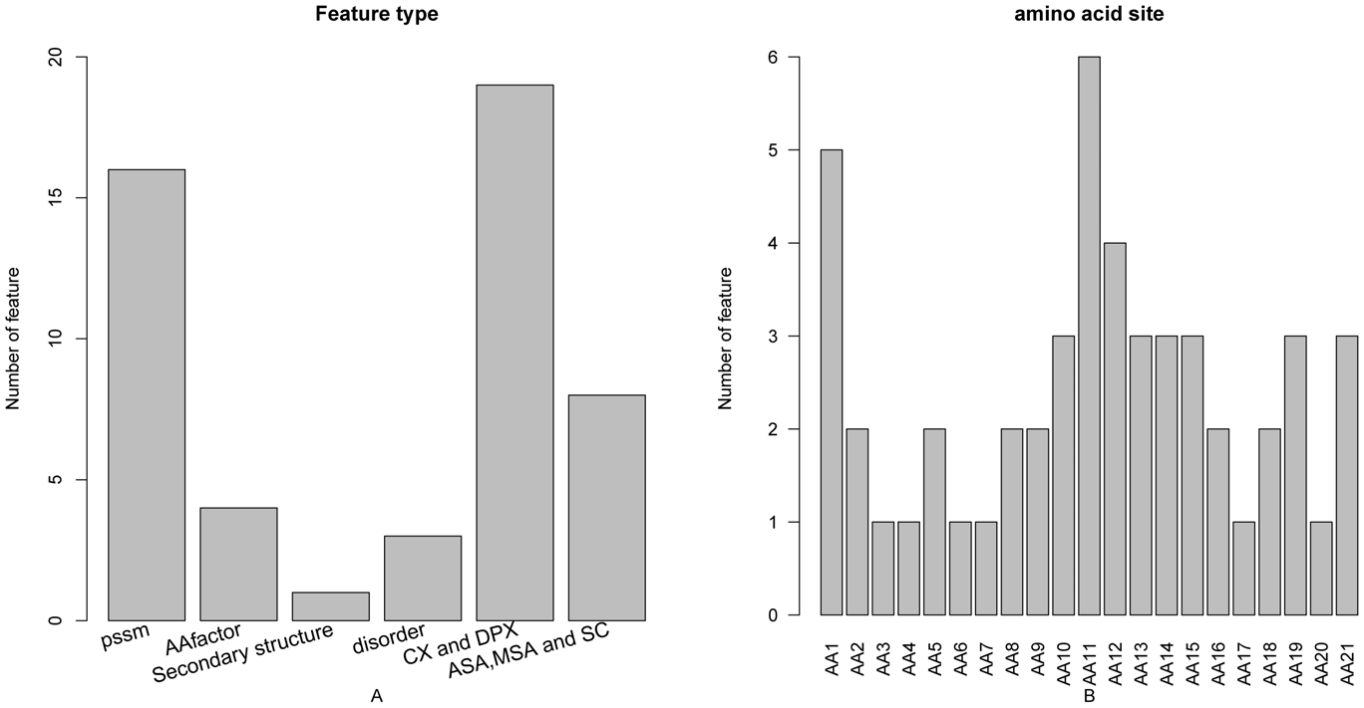
Bar plots to show the feature distribution for the 51 optimal features and the corresponding site distribution. It can be seen from panel A that of the 51 optimal features, 16 were obtained from PSSM conservation scores, 4 from the amino acid factors, 3 from the disorder scores, 1 from the secondary structural propensities, 19 from CX and DPX, and 8 from ASA, MSA and SC. It can be seen from panel B that site 11 played the most important role in determining the PPI sites.

The distribution of the number of each type of feature in the final optimal feature set was investigated and is shown in Fig. 2A. Of the 51 optimal features, 16 were obtained from PSSM conservation scores, 4 from the amino acid factors, 3 from the disorder scores, 1 from the secondary structural propensities, 19 from CX and DPX, and 8 from ASA, MSA and SC. Six kinds of features contributed to the prediction of PPI sites. It was revealed by the site-specific distribution of the optimal feature set (see Fig. 2B) that site 11 played the most important role in determining the PPI sites. In addition, the features of site 1 and site 12 also contribute considerably to the prediction of PPI sites.

### PSSM Conservation Score Feature Analysis

**Figure 3:**
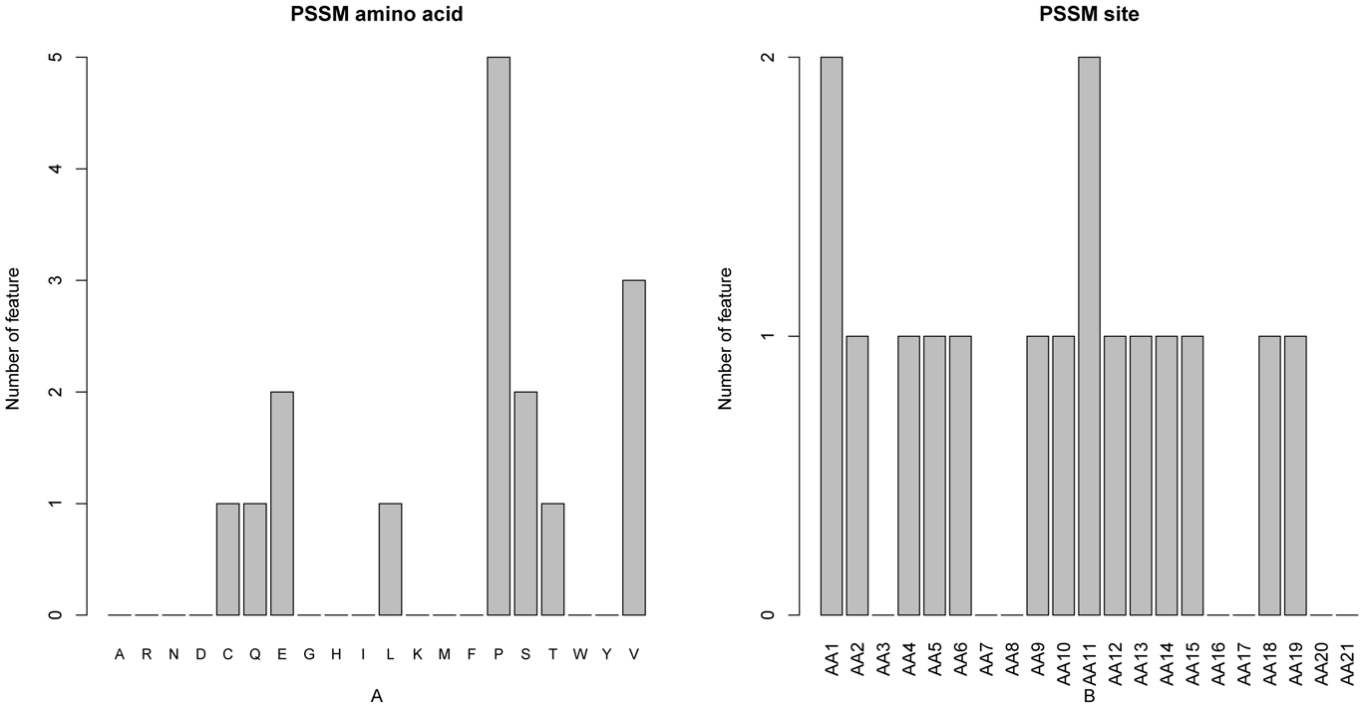
Bar plots to show the distribution in the optimal feature set for the PSSM score and the corresponding specific site score. It can be seen from panel A that the conservation against mutations to amino acid P (Proline) has the most impact on the prediction of PPI sites. It can be seen from panel B that the conservation status at subsites 11 and 1 played the most important role in predicting the PPI site.

As mentioned above, among the 36 optimal features, 16 belonged to the PSSM conservation features, showing the highest proportion. It can be clearly seen from Fig. 3A that each of the 20 different amino acid types has a different number of PSSM conservations in determining the PPI site. In this regard, the conservation status against amino acid P (Proline) contributed most, successively followed by V (Valine) and so forth. Interestingly, it has been reported that P frequently occurs near interaction sites. The unique chemical characteristics of P helps protect the integrity and present the sites, which can promote protein-protein interactions [42]. Unlike other amino acid residues, P can assume either a cis or trans configuration [43]. P disrupts both α-helices and β-sheet conformation owing to the lack of the amide proton and steric hindrance [44], and therefore blocks the propagation of neighboring secondary structures through the interaction site. Thus, P residues form “brackets” on both sides of the interaction sites and help preserve the conformation and integrity of the site which are necessary for molecular recognition and specific interaction [42]. Meanwhile, as shown in Fig. 3B, the conservation status at subsites 11 and 1 played the most important roles in predicting the PPI site, indicating that the conservation status of PPI sites themselves had great influence in determining the PPI sites.

### Amino Acid Factor Analysis

**Figure 4:**
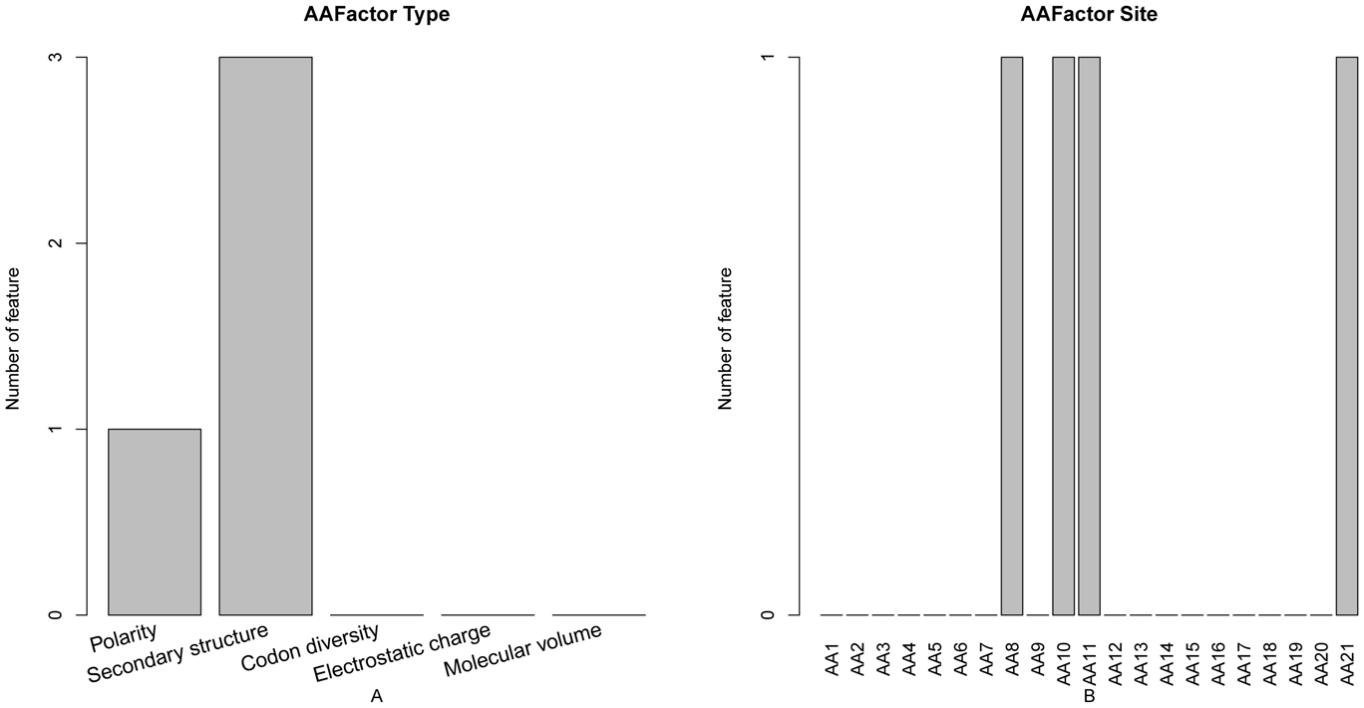
Bar plots to show the distribution in the optimal feature set for the amino acid factor features and the corresponding specific site score. It can be seen from panel A that the secondary structural feature was the most important one for predicting PPI sites. It can be seen from panel B that amino acid residues at subsites 8, 10, 11 and subsite 21 contributed most to PPI site prediction.

Illustrated in Fig. 4 are the contribution points of different amino acid factors and their subsite locations to the PPI site prediction. It can be seen from Fig. 4A that secondary structure was the most important feature in PPI site prediction, which result is supported by the finding that the manner in which protein-protein interactions are formed is determined by the residue type and the secondary structure found in the interface [45]. The secondary structural features appear to be useful for the characterization and classification of PPI sites as reported recently in a study by Guharoy et al. [46]. As shown in Fig. 4B, the amino acid residues at subsites 8, 10, 11 and subsite 21 contributed most to the PPI site prediction. Furthermore, the secondary structural feature of subsite 11 had an index of 9 in the optimal feature set, suggesting that the secondary structure of the PPI site itself was one of the most important features for prediction of the PPI site.

**Figure 5:**
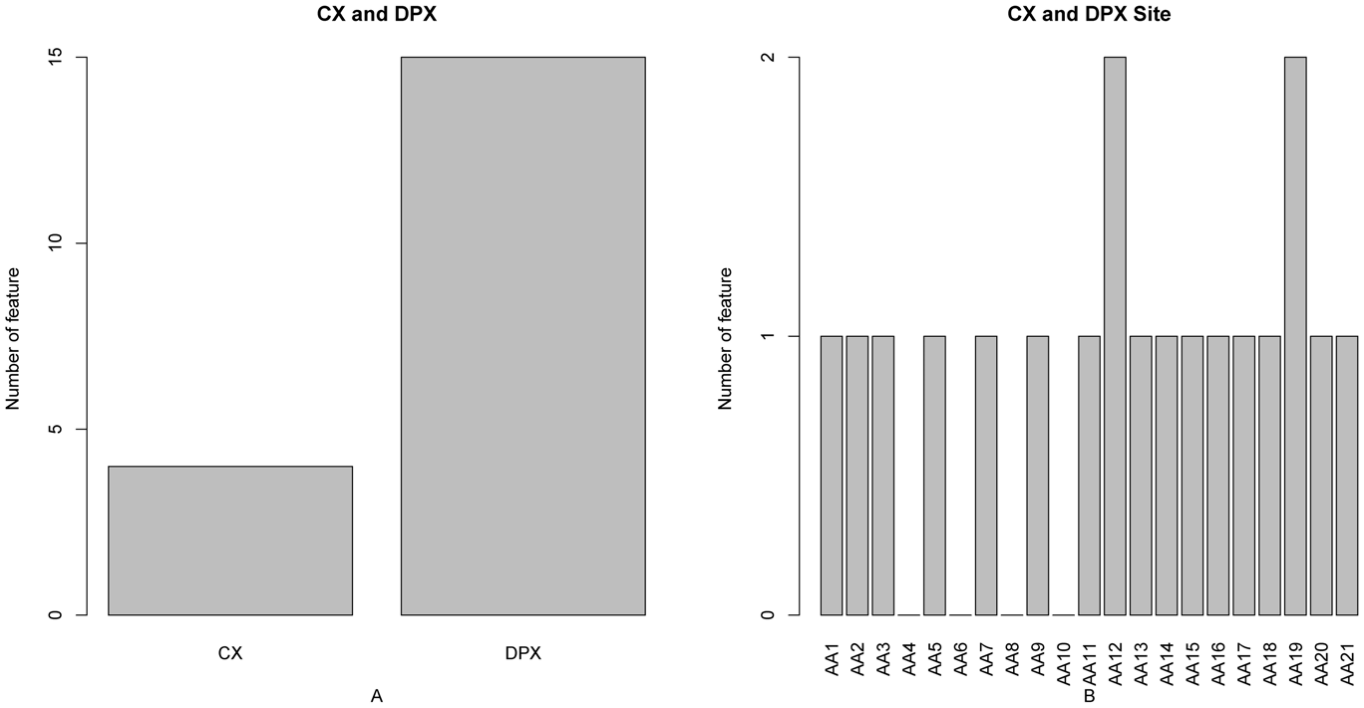
Bar plots to show the distribution in the optimal feature set for the protrusion index and depth index features, and the corresponding specific site score. It can be seen from panel A that both CX and DPX contribute to the prediction of PPI sites, and that DPX has more influence than does CX. It can be seen from panel B that the CX and DPX features at subsites 12 and 19 contribute more to the PPI site prediction.

### Disorder Analysis

Within the final optimal feature set, three disorder features were selected. These three disorder features were from subsites 1, 16 and 21. In particular, the disorder feature of subsite 1 had an index of 1 in the optimal feature set, suggesting that it was one of the most important features in the PPI prediction. The non-regular structures have importance in mediating interactions in interfaces formed by heterocomplexes [46]. In addition, it has been reported that disordered structures as well as helices constitute most of the PPI regions (about 92%) [47]. Also, the disorder feature of subsite 21 has an index of 6 in the final optimal feature site.

**Figure 6:**
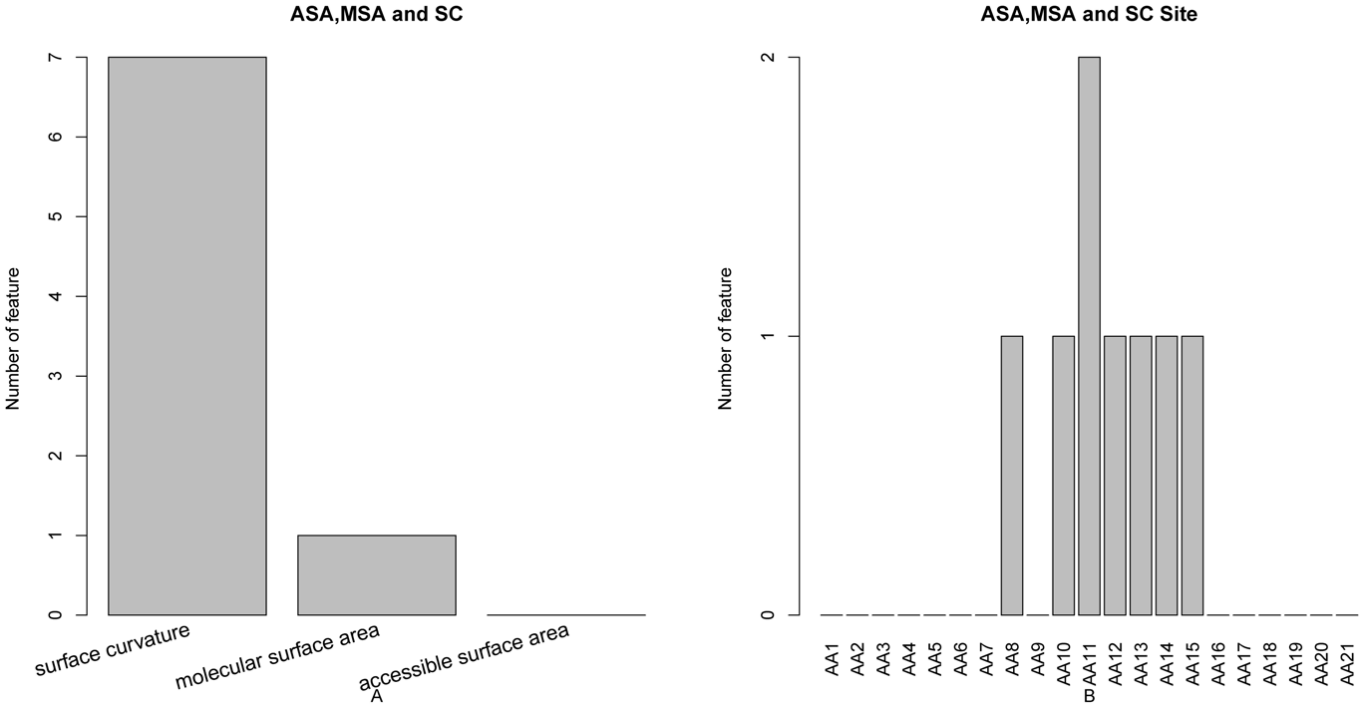
Bar plots to show the distribution in the optimal feature set for the accessible surface area, molecular surface area and surface curvature features, and the corresponding specific site score. It can be seen from panel A that SC plays the most important role in determination of PPI sites as compared with AS and MS. It can be seen from panel B that SC features at subsite 8 and subsites 10–15 contribute relatively more to PPI site prediction.

### Secondary Structural Features Analysis

There was one feature of secondary structure in the optimal feature set. This was other secondary structure besides helix and strand of site 1, which was consistent with the discussion above that PPI regions preferred to have a non-regular structure [46], [47].

**Figure 7:**
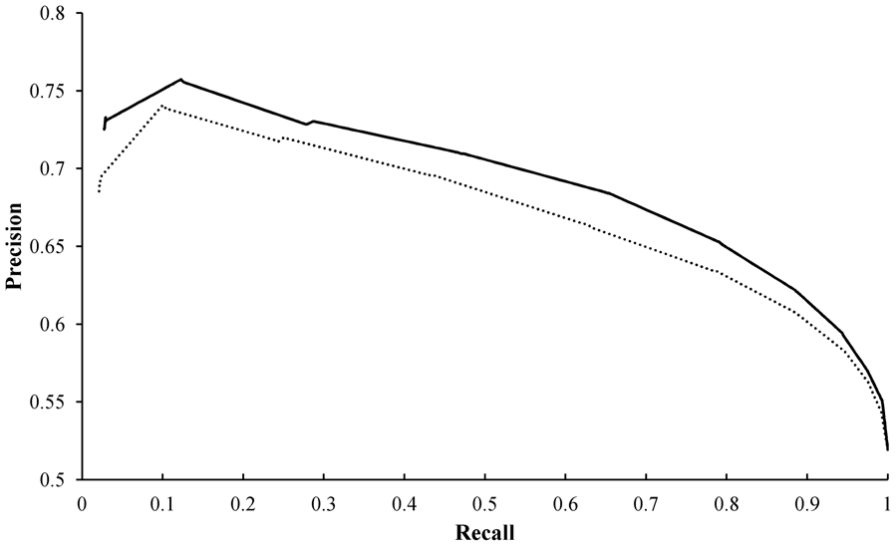
Precision–recall graph for prediction based on sequence and structural features. The figure presents precision–recall curves for the following methods: our method with structural features (solid line) and the method of Sikic et al. [9] with structural features (dashed line).

### Protrusion Index (CX) and Depth Index (DPX) Feature Analysis

Shown in Fig. 5 are the CX and DPX features in the optimal feature set. It can be seen from Fig. 5A that both CX and DPX contribute to the prediction of PPI sites, and DPX has more influence than CX, an observation consistent with the variable importance revealed in a study by Sikic et al. [9]. It was shown that both DPX and CX can contributed to the prediction of PPI sites and that DPX had more influence than CX (Fig. 5 in ref. [9]). Because PPIs are generally dominated by hydrogen bonds, salt bridges and hydrophobic contacts across the interface, complementary protein surface patterns underlying local interactions must be desolvated, densely packed and hence deeply buried to make a contribution to the binding free energy [48]. Compared to other residues, it has been found that PPI sites generally have a larger relative side-chain accessible surface area and a larger average DPX [49]. Moreover, it can be seen from Fig. 5B that the CX and DPX features at subsites 12 and 19 contribute more to the PPI site prediction. There are two DPX features and a CX feature within the top 10 of the optimal feature set, which are DPX at subsite 16 and subsite 1 and CX at subsite 12.

### Accessible Surface Area (AS), Molecular Surface Area (MS) and Surface Curvature (SC) Feature Analysis

Shown in Fig. 6 are the AS, MS and SC features in the optimal feature set. It can be seen from Fig. 6A that SC plays the most important role in determination of PPI sites as compared with AS and MS. PPI sites are widely known to have concave structures on their surfaces because of binding stability, specificity, and reaction promotion [50]. Much research has been conducted into searching for and extracting pockets from the protein surface as candidates of interaction sites [51], [52]. In addition, it has been shown that interface surface characteristics including surface curvature play important roles in protein-DNA interaction [53]. Thus, SC probably also is important for PPI. In addition, It is exactly the saddle-shaped curvatures that give rise to membrane-protein interactions [54]. Therefore, SC can be used as a promising feature for prediction of PPI sites from protein structural data. Moreover, it can be seen from Fig. 6B that the SC features at subsite 8 and subsites 10–15 contribute relatively more to PPI site prediction. There are three SC features in the optimal feature set, which are SC at subsite 11, subsite 14 and subsite 12 with an indices of 2, 5 and 10 respectively. It is suggested that the SC features of the PPI site itself play a key role in the prediction of PPI sites.

### Comparing the Prediction Performances of Different Methods

To determine whether the 3D structural features contribute to the prediction of the PPI site, we constructed another dataset without 3D structural features. Listed in Information S4 are the rates of prediction accuracy, specificity, sensitivity and MCC based on the dataset without 3D structural features. As we can see in Table 1, the prediction accuracy and MCC were better when using 3D structural features (accuracy: 0.672997, MCC: 0.347977) than without (accuracy: 0.596689, MCC: 0.190073). The comparison suggests that the 3D structural features indeed contribute to the prediction of PPI sites.

In addition, we compared our method with that of Sikic et al. [9] on the same dataset used in our study, since their data was not publicly available. The dataset was encoded with features mentioned in their work and features proposed in our study separately. Random forest and 10-fold across-validation were employed to establish predictive models for both methods. As show in Table 1, the accuracy (0.672997) and MCC (0.347977) of our method were better than those of Sikic et al. (0.653001 and 0.307795). In addition, from the Precision-Recall curve in Fig. 7, we can see that the curve of our method with 3D structural features was above that of Sikic et al. Therefore, the features proposed in our study appear more effective for prediction of PPI sites.

### Directions for Experimental Validation

The selected features at different sites may provide clue for researchers to use in finding or validating new determinants of PPI sites. For example, we reveal that proline(P) plays a pivotal role in PPI site determination, consistent with the report that P frequently occurs near interaction sites [42]. In addition, we highlight the importance of secondary structure, in agreement with previous studies [45], [46]. There were three disorder features in the optimal feature set, indicating that disorder features are important for prediction of PPI sites, consistent with the notions that the non-regular structures have importance in mediating interactions in interfaces formed by heterocomplexes [46] and that disordered structures as well as helices constitute most of the PPI regions (about 92%) [47]. The important role of DPX in prediction of PPI sites revealed in our study has been supported by previous studies [9], [49]. It was revealed in our study that surface curvature plays an important role in determination of PPI sites, whose role in protein-DNA interaction has been confirmed [53]. Thus, the remaining features in the optimal feature set are seen to be worthy of validation by experiments and further research.

### Conclusion

In this study, we developed a new method for the prediction of PPI sites. Our method considers not only the physicochemical features of each amino acid but also the sequence conservation information and residue disorder status within the PPI region. In addition, we also took into consideration the solvent accessibility, secondary structure of amino acids in the PPI region and 3D structural features from PDB data. Our approach achieved an overall MCC of 0.347977 with 51 features. We also show that the accuracy of the classification can be improved through the use of 3D structural information. On the basis of the feature selection algorithm, an optimal set of features were selected, which are regarded as the features that contribute most significantly to the prediction of PPI sites. The selected features may shed some light on the mechanism of PPI and provide guidelines for experimental validation.

## Supporting Information

Information S1 This file contains two sheets. The first one shows the MaxRel feature table, which ranked the 714 features according to the relevance between features and class of the samples. The second one shows the mRMR feature table, which ranked the 714 features according to the redundancy and relevance criteria.
(XLSX)

Information S2 The sensitivity (Sn), specificity (Sp), accuracy (Ac), Matthews correlation coefficient (MCC) of each runs of IFS for the dataset with 3D structural features. The IFS curve was plotted based on this file.
(XLSX)

Information S3 The 51 features selected by the IFS procedure.
(XLSX)

Information S4 The sensitivity (Sn), specificity (Sp), accuracy (Ac), Matthews’s correlation coefficient (MCC) of each run of IFS for the dataset without 3D structural features. The IFS curve was plotted based on this file.
(XLSX)
